# Exosomes from Human Umbilical Cord Mesenchymal Stem Cells: Identification, Purification, and Biological Characteristics

**DOI:** 10.1155/2016/1929536

**Published:** 2016-12-25

**Authors:** Bin Zhang, Li Shen, Hui Shi, Zhaoji Pan, Lijun Wu, Yongmin Yan, Xu Zhang, Fei Mao, Hui Qian, Wenrong Xu

**Affiliations:** ^1^Key Laboratory of Laboratory Medicine of Jiangsu Province, School of Medicine, Jiangsu University, Zhenjiang, Jiangsu, China; ^2^The Affiliated Hospital, Jiangsu University, Zhenjiang, Jiangsu 212000, China

## Abstract

Our and other groups have discovered that mesenchymal stem cells (MSCs) derived exosomes are a novel therapeutical modality for many diseases. In this study, we summarized a method to extract and purify hucMSCs-exosomes using ultrafiltration and gradient centrifugation in our laboratory and proved that hucMSCs-exosomes prepared according to our procedure were stable and bioactive. Results showed that exosomes derived from hucMSC were 40~100 nm and CD9 and CD81 positive. Functionally, hucMSCs-exosomes promoted cell proliferation and protected against oxidative stress-induced cell apoptosis* in vitro* by activation of ERK1/2 and p38. Interestingly, UV exposure abrogated the regulatory roles of exosomes under oxidative stress, indicating that hucMSCs-exosomes may regulate cell growth and apoptosis by exosomal shuttle of RNA. Furthermore, cytokine profile analysis revealed that hucMSCs-exosomes contained high dose of IL-6, IL-8, and other cytokines. The established method is practical and efficient, which provides a basis for further evaluating the potential of hucMSCs-exosomes as therapeutic agents.

## 1. Introduction

Exosomes are 40–100 nm extracellular membrane vesicles of endocytic origin, which were firstly discovered in the early 1980s [1–3]. Exosomes are released into the extracellular environment upon fusion of multivesicular bodies with the plasma membrane [2, 4–6]. Exosomes are secreted by most cells that have been examined so far, including mast cells, dendritic cells [7, 8], B cells [6], T cells [9], tumor cells [10, 11], and epithelial cells. In addition, exosomes have been found in many biological fluids [1, 11–16] including plasma [12], urine [13], saliva [14], and breast milk [15].

It has been shown that the exosomal protein composition depends on the cellular source of the studied exosomes. Regardless of origin, several common proteins are found in exosomes, including chaperones, cytoskeletal proteins, and tetraspanins such as CD9, CD63, and CD81 [17–20]. Furthermore, more studies have indicated that exosomes also contain a substantial amount of small molecules that can be transferred from one cell to another. Exosomes could easily communicate with target cells through specific receptor-ligand interactions and shuttle defined patterns of components such as proteins, bioactive lipids, and RNA to induce biological effects [19, 21–24]. Therefore, many investigations have been performed to demonstrate the role of exosomes in paracrine/endocrine process and genetic information exchange between different cells due to their important bioactivity in tissue microenvironment [21, 22].

Increasing studies have pointed out the potential contribution of human mesenchymal stem cells in the recovery of different types of tissue injury [25–28]. Although it has been demonstrated that MSCs mediate tissue repair through paracrine and transdifferentiation mechanisms, the details responsible for their roles are not well understood. Our recent studies showed that exosomes derived from human umbilical cord MSCs (hucMSCs) alleviate CCl4-induced liver fibrosis, enhance cutaneous wound healing, and repair cisplatin-induced acute kidney injury (AKI) [29–33]. In these studies, we established a method to extract and purify exosomes from hucMSCs with ultrafiltration and gradient centrifugation. Herein, we uncovered more detailed information about this method and other functions of hucMSC-exosome* in vitro*.

## 2. Materials and Methods

### 2.1. Isolation and Purification of Exosomes

HucMSCs were isolated as previously described in our work [34]. All experiment protocols were approved by the Ethics Committee of Jiangsu University. The 70%–80% of the confluent hucMSCs cultures were washed twice with phosphate-buffered saline (PBS) and then incubated in serum-free low glucose Dulbecco's modified Eagle's medium (LG-DMEM) for 48 hours. The conditioned medium was collected and centrifuged at 1,000 ×g for 20 minutes to remove cell debris, followed by centrifugation at 2,000 ×g for 20 minutes and 10,000 ×g for 20 minutes. The supernatant was collected and concentrated using 100 KDa MWCO (Millipore, USA) at 1,000 ×g for 30 minutes. The concentrated supernatant was loaded upon 5 mL of 30% sucrose/D_2_O cushions and then ultracentrifuged at 100,000 ×g for 60 minutes (optimal-90K, Beckman Coulter). The microvesicles-enriched fraction was harvested and diluted with PBS and then centrifuged thrice at 1,000 ×g for 30 minutes using 100 KDa MWCO. Finally, the purified exosomes were collected and subjected to filtration on 0.22 *μ*m pore filter (Millipore, USA) and stored at −70°C.

### 2.2. Transmission Electron Microscopy

20 *μ*L drops of purified exosomes were adsorbed onto copper grids, placed for 1 minute at room temperature, adsorbed onto the superfluous exosomes, and stained with 30 g/L phosphotungstic acid (pH 6.8) for 5 minutes at room temperature; the sample dried under half-watt lamp. Samples were imaged using a transmission electron microscopy (FEI Tecnai 12, Philips).

### 2.3. SDS-PAGE Analysis and Western Blotting

For SDS-PAGE analysis, total proteins in hucMSCs and exosomes were separated on 12% SDS-polyacrylamide gels and stained with Coomassie Blue. For Western blot assay, the proteins were electroblotted onto a nitrocellulose membrane after separating on 12% SDS-PAGE. The membrane was blocked and incubated with the primary antibodies followed by incubation with the horseradish peroxidase-coupled secondary antibody. The bands were visualized with ECL plus system from Amersham Pharmacia Biotech (Buckinghamshire, UK). The sources of primary antibodies were as follows: CD9 (Bioworld Technology, USA), CD81 (Epitomics, USA), *β*-actin (Bioworld Technology, USA), p-ERK1/2, T-ERK1/2, p-P38, P38 (Santa Cruz Biotechnology, USA), PCNA (Bioworld Technology, USA), and GAPDH (Shanghai KangChen Biotechnology, China).

### 2.4. Cell Culture and Oxidative Stress Treatment

H9C2(2-1) cells were cultured in HG-DMEM with 10% fetal bovine serum (FBS, Gibco, USA). HL-7702 cells were cultured in RPMI-1640 with 20% NBS. NRK-52E cells were cultured in HG-DMEM with 10% NBS. To induce oxidative stress, H9C2(2-1), HL-7702, and NRK-52E cells were exposed to 300, 300, and 500 *μ*M H_2_O_2_ for 24, 6, and 6 hours, respectively. H9C2(2-1), HL-7702, and NRK-52E cell lines were all bought from the Cell Bank of the Chinese Academy of Sciences, Shanghai.

### 2.5. Cell Viability

Cell viability was assessed by MTT assay (*n* = 5). 1 × 10^3^ cells were seeded per well under normal condition and 5 × 10^3^ cells were seeded per well under oxidative condition in 96-well plate, respectively. Then, cells were treated with different doses of hucMSCs-exosomes (100 and 800 *μ*g/mL) for 24 and 48 hours under normal condition or pretreated with hucMSCs-exosomes (800 *μ*g/mL) for 24 hours under oxidative condition. After incubation, the absorbance was measured at 570 nm using a microplate reader.

### 2.6. Cell Apoptosis

Cell apoptosis was evaluated using a terminal deoxynucleotidyl transferase mediated dUTP nick end labeling (TUNEL) assay and mitochondria membrane electric potential assay. The TUNEL assay was performed using an* in situ* cell death detection kit (Boster Bioengineering, Wuhan, China) according to the manufacturer's instructions. TUNEL-positive apoptotic cells were counted in 10 consecutive fields in the slides. The mitochondria membrane electric potential was performed using a JC-1 detection kit (Beyotime Institute of Biotechnology, Shanghai, China) according to the manufacturer's instructions. The fluorescent signal was observed under a fluorescence microscopy.

### 2.7. Immunohistochemistry

Immunohistochemical staining of proliferative cell nuclear antigen (PCNA) was performed using an SABC immunohistochemistry detection kit (Boster Bioengineering, Wuhan, China) according to the manufacturer's instructions. The cells were fixed, blocked, and incubated with the primary antibody (1 : 200) for 2 hours followed by the secondary antibody for 1 hour. PCNA-positive cells were counted in 10 consecutive fields in the slides.

### 2.8. UV Exposure

HucMSCs-exosomes were subjected to UV exposure (254 nm) for 1 hour at 4°C [35, 36]. The control was kept at 4°C for 1 hour without UV exposure. These two exosomes were then added to the cells at the concentration of 800 *μ*g/mL and the cells were cultured under H_2_O_2_-induced oxidative stress.

### 2.9. Cytokine Array

The profile and concentration of cytokines in hucMSCs-exosomes and the conditioned medium of hucMSCs were quantified using Luminex assay.

### 2.10. Statistical Analysis

Data was presented as mean ± SD. Statistical variance was analyzed by ANOVA using Prism software (Graph Pad, San Diego, USA). Statistical *p* values less than 0.05 were considered significant.

## 3. Results

### 3.1. Characterization of HucMSCs-Exosomes

**Figure 1:**
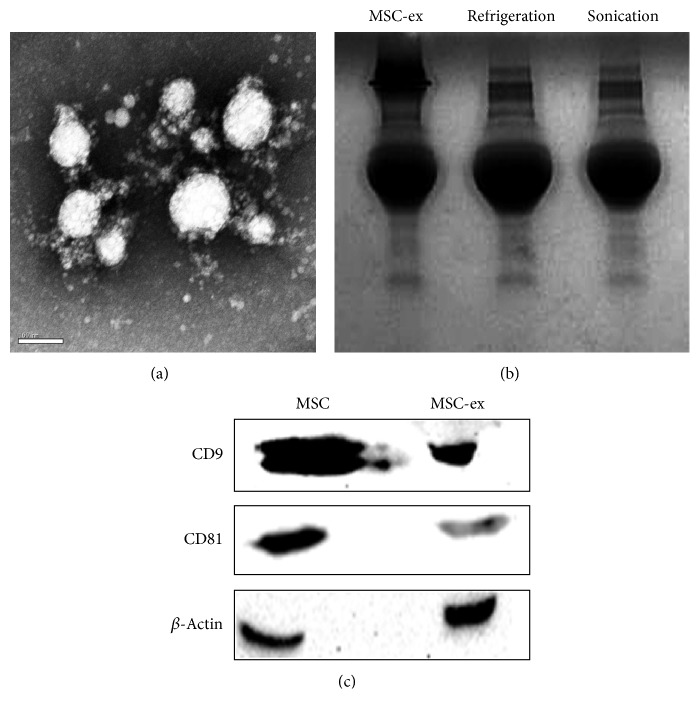
Characterization of exosomes from hucMSCs. (a) Transmission electron microscopy analysis of extracellular vesicles secreted by hucMSCs. Scale bar: 100 nm. (b) Coomassie Blue staining. The protein extracts of hucMSCs-exosomes were separated by SDS-PAGE. (c) Western blotting analyses of exosomal markers using antibodies against CD9 and CD81.

Transmission electron microscopy analysis showed a spheroid morphology of the purified exosomes, with a mean diameter of 40–100 nm (Figure 1(a)). The protein extract of exosomes was separated on 12% SDS-PAGE gel and stained with Coomassie Blue. As shown in Figure 1(b), the extract of exosomes enriched proteins with molecular weight ranging from 55 to 72 KDa and this enrichment was not affected by refrigeration or sonication. Equal amounts of protein extracts from hucMSCs and hucMSCs-exosomes were analyzed by Western blotting using antibodies specific to CD9 and CD81, which were exosomal markers. The results showed that CD9 and CD81 were constitutively expressed in hucMSCs-exosomes (Figure 1(c)). Together, these results indicate that we have successfully isolated and identified exosomes from the extracellular medium of hucMSCs.

### 3.2. HucMSCs-Exosomes Stimulate Cell Proliferation* In Vitro*

**Figure 2:**
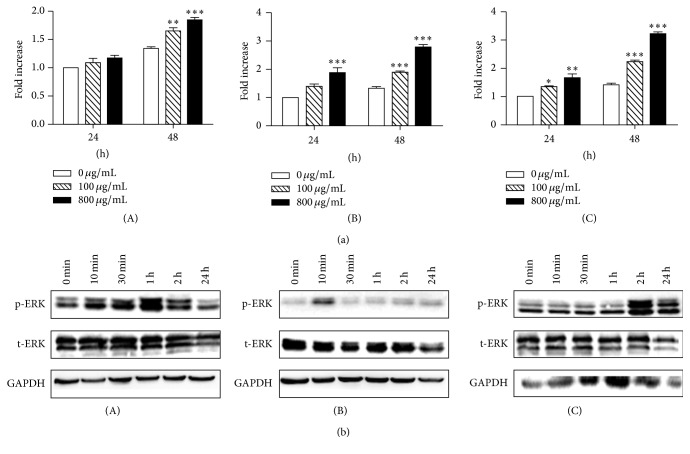
HucMSCs-exosomes promote cell proliferation* in vitro*. (a) HucMSCs-exosomes promote cell proliferation in a dose- and time-dependent manner. Results were shown as mean ± SD (*n* = 5). ^*∗*^ *p* < 0.05, ^*∗∗*^ *p* < 0.01, and ^*∗∗∗*^ *p* < 0.001 compared to control group. (b) Western blotting analysis of phosphorylated ERK1/2 in cells treated with 800 *μ*g/mL hucMSCs-exosomes as indicated. (A) H9C2(2-1); (B) HL-7702; and (C) NRK-52E.

Incubation of H9C2(2-1), HL-7702, and NRK-52E cells with hucMSCs-exosomes promoted cell proliferation in a dose- and time-dependent manner compared to the control cells which were incubated with vehicle alone (conditioned medium) (Figure 2(a)). To further demonstrate the signaling pathway regulated by exosomes, we detected the levels of total and phosphorylated ERK1/2 as this pathway is tightly linked with cell growth. The results showed that exosomes treatment resulted in an increase in the phosphorylation of ERK1/2 (Figure 2(b)), suggesting that hucMSCs-exosomes may promote cell growth through upregulation of ERK1/2 phosphorylation.

### 3.3. HucMSCs-Exosomes Protect Cell Viability* In Vitro*

**Figure 3:**
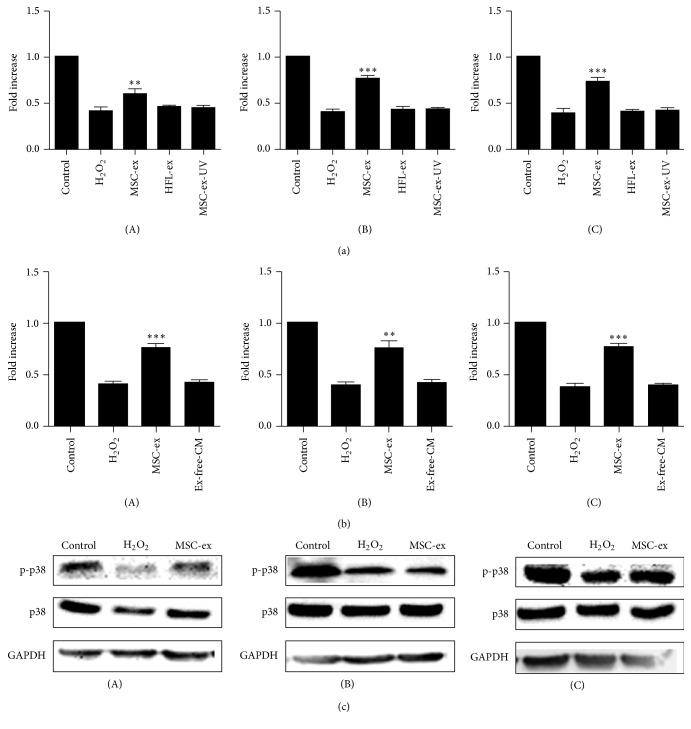
HucMSCs-exosomes protect cell viability* in vitro*. (a) MTT assay. H9C2(2-1), HL-7702, and NRK-52E cells were pretreated with different sources of hucMSCs-exosomes (800 *μ*g/mL) for 24 hours, followed by exposure to H_2_O_2 _(300 *μ*M) for 24 hours. Results were shown as mean ± SD (*n* = 5). ^*∗∗*^ *p* < 0.01 and ^*∗∗∗*^ *p* < 0.001 compared to H_2_O_2_ group. (b) MTT assay. H9C2(2-1), HL-7702, and NRK-52E cells were pretreated with different sources of hucMSCs-exosomes (800 *μ*g/mL) or exosomes-free conditioned medium collected from hucMSCs (EX-free-CM) for 24 hours, followed by exposure to H_2_O_2_ (300 *μ*M) for 24 hours. Results were shown as mean ± SD (*n* = 5). ^*∗∗*^ *p* < 0.01 and ^*∗∗∗*^ *p* < 0.001 compared to H_2_O_2_ group. (c) Western blotting analysis of phosphorylated p38. H9C2(2-1), HL-7702, and NRK-52E cells were pretreated with hucMSCs-exosomes (800 *μ*g/mL) followed by exposure to H_2_O_2 _as described above.

Oxidative stress induced by H_2_O_2_ leads to loss of cell viability* in vitro*. We further test if hucMSCs-exosomes promoted cell survival under H_2_O_2_-induced oxidative stress. Exosomes that derived from different sources were added to the cells for 24 hours, and then the cells were exposed to oxidative stress for different periods of time and the cell viability was examined by MTT assay. As shown in Figure 3(a), H_2_O_2_ decreased the viability of cells, but hucMSCs-exosomes increased the percentage of cell viability compared to H_2_O_2_ group, suggesting that exosomes' pretreatment antagonizes H_2_O_2_-induced cell death. And we also found that exosome-free conditioned medium did not show the repair role as hucMSC-exosome and could not reverse the H_2_O_2_-induced inhibition of proliferation (Figure 3(b)). We confirmed that this protective role was specific to hucMSCs-exosomes as the exosomes from human fibroblast-like cells (the Cell Bank of the Chinese Academy of Sciences, Shanghai) (HFL-exosomes) did not have the protective role. Exosomal shuttle of RNA is critical for the function of exosomes. To test if the exosomes-mediated cell protection was due to the transfer of RNA to injured cells, we exposed exosomes to UV (254 nm) for 1 hour as UV exposure inactivates RNA functions. The results showed that the exosomes that exposed to UV lost their protective effects on the cell viability (Figure 3(a)). We further demonstrated that H_2_O_2_ treatment inhibited the phosphorylation of p38 in the cells, but pretreatment with exosomes restored the level of phosphorylated p38 (Figure 3(b)).

### 3.4. HucMSCs-Exosomes Promote Cell Proliferation under Oxidative Stress

**Figure 4:**
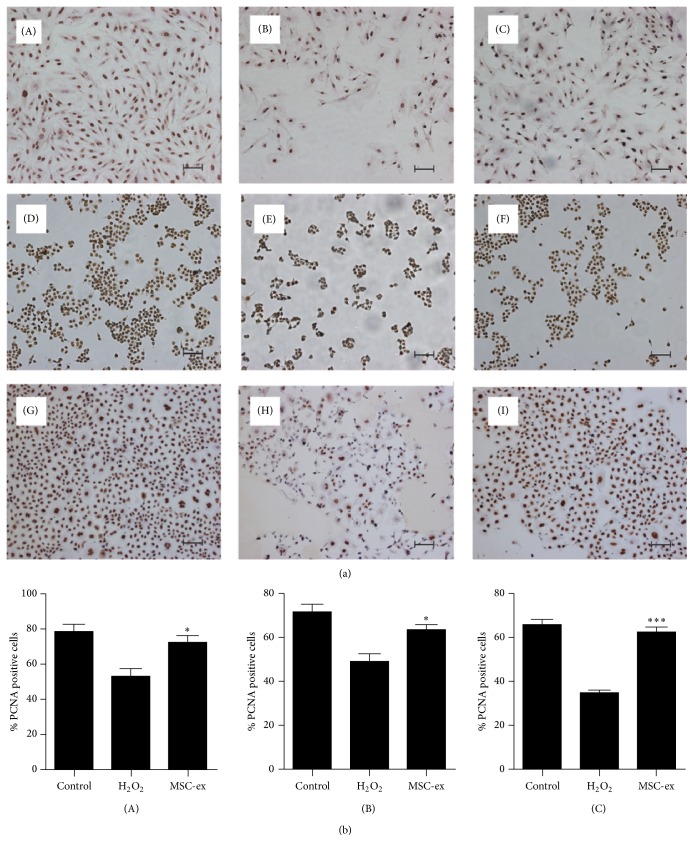
HucMSCs-exosomes promote cell proliferation under oxidative stress. (a) Immunohistochemical staining of proliferative cell nuclear antigen. H9C2(2-1), HL-7702, and NRK-52E cells were pretreated with hucMSCs-exosomes (800 *μ*g/mL) for 24 hours, followed by exposure to H_2_O_2 _(300, 300, and 500 *μ*M) for different time points (24, 6, and 6 hours). (A–C) H9C2(2-1); (D–F) HL-7702; and (G–I) NRK-52E. Control (A, D, and G); H_2_O_2_ (B, E, and H); and hucMSCs-exosomes (C, F, and I). Scale bar: 100 *μ*m. (b) Quantitative analysis of the percentage of PCNA-positive cells.^*∗*^ *p* < 0.05 and ^*∗∗∗*^ *p* < 0.001 compared to H_2_O_2_ group.

We further confirmed the protective role of hucMSCs-exosomes on cell viability by immunohistochemical staining of proliferative cell nuclear antigen (PCNA). HucMSCs-exosomes were added to the cells for 24 hours, and then the cells were exposed to oxidative stress for different periods of time. The results showed that H_2_O_2_ treatment inhibited the percentage of proliferative cells, while exosomes' treatment abrogated this effect (Figures 4(a) and 4(b)).

### 3.5. HucMSCs-Exosomes Inhibit H_2_O_2_-Induced Apoptosis* In Vitro*

**Figure 5:**
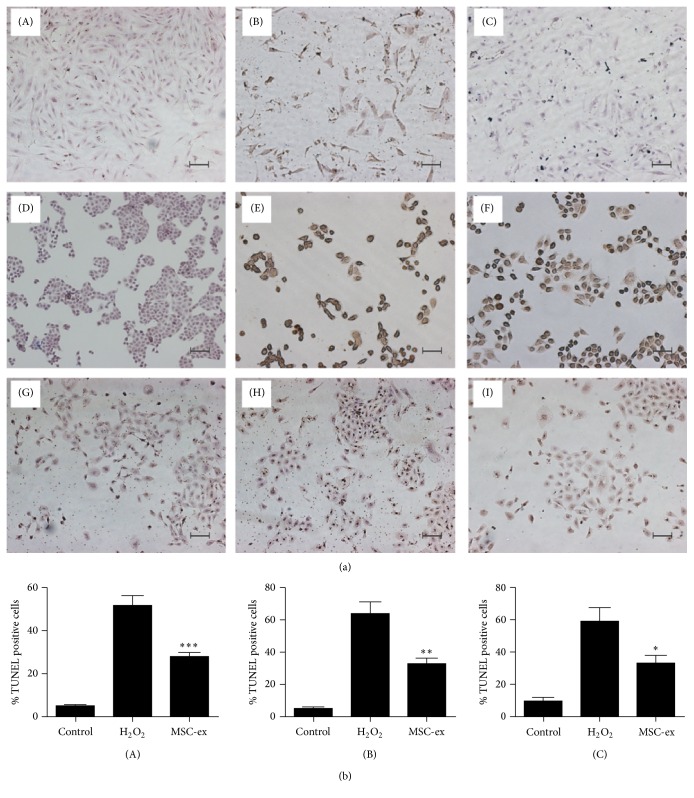
HucMSCs-exosomes inhibit H_2_O_2_-induced apoptosis. (a) TUNEL staining. H9C2(2-1), HL-7702, and NRK-52E cells were pretreated with hucMSCs-exosomes (800 *μ*g/mL) for 24 hours, followed by exposure to H_2_O_2 _(300, 300, and 500 *μ*M) for different time points (24, 6, and 6 hours). (A–C) H9C2(2-1); (D–F) HL-7702; and (G–I) NRK-52E. Control (A, D, and G); H_2_O_2_ (B, E, and H); hucMSCs-exosomes (C, F, and I). Scale bar: 100 *μ*m. (b) Quantitative analysis of the percentage of TUNEL-positive cells. ^*∗*^ *p* < 0.05 compared to H_2_O_2_ group.

**Figure 6:**
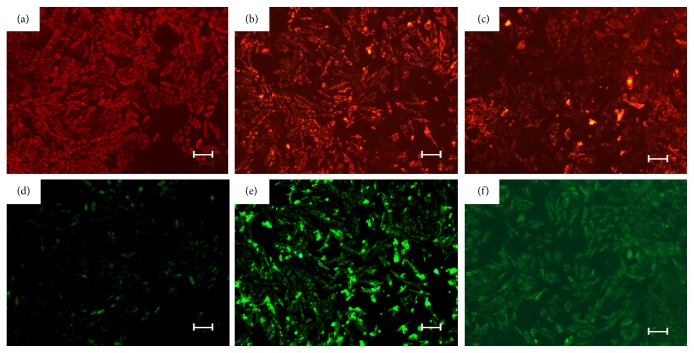
HucMSCs-exosomes inhibit H_2_O_2_-induced mitochondria activation. H9C2(2-1) were pretreated with hucMSCs-exosomes (800 *μ*g/mL) for 24 hours, followed by exposure to H_2_O_2 _(300 *μ*M) for 24 hours. The cells were subjected to JC-1 staining for 15 min. The signals were observed under fluorescence microscope. Control (a, d); H_2_O_2 _(b, e); and hucMSCs-exosomes (c, f).

TUNEL staining showed that H_2_O_2_ treatment resulted in an increase in the percentage of apoptotic cells, but pretreatment with hucMSCs-exosomes decreased the percentage of apoptotic cells to a less extent (Figures 5(a) and 5(b)), indicating that hucMSCs-exosomes inhibited H_2_O_2_-induced apoptosis* in vitro.* To further demonstrate if exosomes regulated cell apoptosis pathways, we performed mitochondria membrane electric potential analysis and the results showed that H_2_O_2_ treatment increased the mitochondria membrane electric potential of H9C2(2-1), HL-7702, and NRK-52E cells (results not shown), while hucMSCs-exosomes treatment decreased the mitochondria membrane electric potential compared to that of H_2_O_2_ group (Figure 6). Collectively, hucMSCs-exosomes inhibit H_2_O_2_-induced apoptosis through regulating mitochondria-mediated cell apoptosis pathway.

### 3.6. Cytokine Profile of HucMSCs-Exosomes

Exosomes contain certain types of cytokines which may mediate its function. We then determined the concentration of cytokines in hucMSCs-exosomes and the conditioned medium of hucMSCs by Luminex assay (*n* = 3). The results showed that hucMSCs-exosomes expressed a lot of cytokines including GM-CSF, IL-15, IL-6, IL-8, TNF-*α*, IL-1*β*, IL-2, and IL-10 (Table 1), in which IL-6 and IL-8 were present in high dose (>100 pg/mL).

## 4. Discussion

In this study, we have isolated and identified exosomes from human umbilical cord MSCs in terms of their biophysical and biological properties: (1) floating at a cushion of 30% sucrose that is a specific attribute of exosomes; (2) nanometer-sized distribution of exosomes (40–100 nm); and (3) exosomal protein expression (CD9 and CD81) [17–20]. Our results confirmed that the microvesicles we isolated from the extracellular medium of hucMSCs were exosomes. The method we established is a practical and efficient procedure to isolate and purify exosomes from hucMSCs.

The exact functions of exosomes are not yet fully understood, although exosomes harvested from different cells have been shown to mediate a multitude of biological effects, including antigen presentation, induction of apoptosis, and promotion of cancer cell growth [6, 37–42]. It seems that the exosomes from different cells have various functions. In the current study, we demonstrated that exosomes derived from hucMSCs had stimulatory effect on cell proliferation and protective role against H_2_O_2_-induced cell death [43], suggesting that the exosomes from hucMSCs prepared using our procedure are bioactive and have supportive role in the growth of cells such as cardiomyocytes, hepatocytes, and renal cells.

The work from different laboratories has indicated that the release of exosomes is a mechanism by which cells transfer material and signals to other cells. Exosomes are round membrane vesicles. The nucleic acid and proteins in the exosomes are protected by the membrane structure. Exosomes have a particular composition reflecting their origin and can transfer not only membrane components but also nucleic acid between different cells. UV exposure of exosomes abrogated their effects on preventing oxidative stress-induced cell death, suggesting that the RNA shuttled by exosomes is one of the critical effectors of their biological effects. We also verify that the exosomes contain several types of cytokines, in which IL-6 and IL-8 are the major ones. Further studies are required to define the physiological role of these cytokines in hucMSCs-exosomes.

The advantage of using exosomes in regenerative medicine rather than the stem cells themselves is avoidance of possible long-term pathologic differentiation of engrafted cells. Compared to cell-based therapies, non-cell-based therapies are generally easier to manufacture and safer as they are nonviable and do not elicit immune rejection. Therefore, the isolation and identification of exosomes from hucMSCs provide a novel approach to treat diseases such as myocardial ischemia/reperfusion injury.

In addition to their tissue regenerative capacity, MSCs and their exosome also display immune-modulatory properties [44, 45]. MSCs constitutively express low levels of major histocompatibility complex-I molecules and do not express costimulatory molecules such as CD80, CD86, or CD40, thus lacking immunogenicity [46]. Based on these properties, MSCs are being used in the treatment of autoimmune diseases and graft-versus-host disease. The earliest indications of the immunosuppressive nature of MSC were derived from studies with human, baboon, and murine MSC demonstrating that MSCs were able to suppress T-lymphocyte activation and proliferation* in vitro* [46–49]. MSCs inhibit immunoglobulin production and arrest B-lymphocytes in the G0/G1 phase of the cell cycle [50]. In conclusion, MSCs possess a remarkably diverse array of immunosuppressive characteristics [51]. MSCs-exosomes might play a different role in immune-modulating activities and mechanisms. Rahman et al. reported that abnormal or excess exosomes released by these MSC-like precursor cells in islets may trigger tissue-specific autoimmunity in the NOD mouse strain [52]. MSC-derived extracellular vesicles also prevent postischemic immunosuppression [45]. These two studies hold the review that MSC-exosome can enhance the activation of immunity. However, other groups believed that MSC-exosome inhibited the inflammatory response to induce tissue regeneration. MSCs-derived exosome suppressed the secretion of proinflammatory factors TNF-*α* and IL-1*β* but increased the concentration of anti-inflammatory factor TGF-*β* [53]. Zhang et al. considered that MSC-secreted exosome has the potential to attenuate an activated immune system through the induction of anti-inflammatory cytokines and Tregs [54]. The aforementioned studies show that immune regulation may also be an important mechanism of exosome in tissue regeneration.

In conclusion, in this study, we have established a practical and efficient method to isolate and identify exosomes from human umbilical cord stem cells and demonstrated the role of hucMSCs-exosomes in stimulating cell proliferation and protecting against oxidative stress-induced apoptosis. Our work provides a basis for further evaluating the potential of hucMSCs-exosomes as therapeutic agents.

## Figures and Tables

**Table 1:** Identification of cytokine from hucMSCs-exosomes and hucMSCs using Luminex.

Cytokine name	HucMSCs-exosomes (pg/mL)	HucMSCs (pg/mL)
GM-CSF	1.75	8.94
IL-15	4.02	0.51
IL-17	<0	<0
IL-6	123.57	649.96
IL-8	285.26	5024.88
TNF-*α*	0.02	0.11
IL-*β*	0.05	0.31
IL-2	0.06	0.21
EGF	<0	3.54
IL-10	0.74	0.73
VEGF	<0	13.65
